# Shenfu Injection Adjunct with Platinum-Based Chemotherapy for the Treatment of Advanced Non-Small-Cell Lung Cancer: A Meta-Analysis and Systematic Review

**DOI:** 10.1155/2017/1068751

**Published:** 2017-11-02

**Authors:** Ailing Cao, Hailang He, Mengxin Jing, Beibei Yu, Xianmei Zhou

**Affiliations:** Department of Respiratory Medicine, Affiliated Hospital of Nanjing University of Chinese Medicine, 155 Hanzhong Road, Nanjing 210009, China

## Abstract

Platinum-based chemotherapy is one of the standard treatments for non-small-cell lung cancer (NSCLC), while its high toxicity and limited clinical effects raise big concerns. Shenfu injection (SFI) has been commonly used as an adjutant chemotherapy drug for NSCLC in China. We ascertained the beneficial and adverse effects of SFI in combination with platinum-based chemotherapy for advanced NSCLC by using meta-analysis methods. The randomized controlled trials (RCTs) involving advanced NSCLC treatment with SFI plus platinum-based chemotherapy versus chemotherapy alone were searched on 6 medical databases up to February 2017. Cochrane handbook 5.1.0 was applied to assess the quality of included trials and RevMan 5.3 software was employed for data analysis. 23 RCTs including 1574 patients met our inclusion criteria. We evaluated the following outcome measures: objective tumor response (ORR), disease control rate (DCR), Karnofsky performance score (KPS), adverse effects, and indicators of cellular immune function. The meta-analysis indicated that SFI plus platinum-based chemotherapy may benefit the patients with NSCLC on attenuated synergies of chemotherapy. These findings need to be confirmed by further rigorously designed high-quality and large-scale RCTs.

## 1. Introduction

Lung cancer is the leading cause of cancer-related mortality and is responsible for 1.38 million deaths each year [1]. Its 5-year survival rate remains low at 15% for patients, which is poor when compared to other high incidence cancers [2]. Reports from previous work have indicated that lung cancer is the most common cancer among both men and women that carries tremendous social and economic burden in both developed and developing countries [3].

NSCLC is the most common form of lung cancer, accounting for approximately 85% of all cases [4]. Over 50% of patients with NSCLC have reached advanced stages at the time of their initial diagnosis due to its high rate of malignancy and invasion. Hence, they are forced to accept other standard therapies, such as chemotherapy, radiotherapy, targeted therapy, or immunotherapy for unresectable tumors [5–7]. Platinum-based chemotherapy, as an optimal and potentially curable treatment for advanced NSCLC patients, occupies the dominant position in the nonsurgical treatment and has made favorable efficacy for reducing tumor size. However, despite the technological advances, the toxicity of chemotherapy is remarkable, which may lead to limited clinical efficacy, substandard immune function, and poor quality of life for patients. Many patients have difficulty completing the recommended number of cycles and thus miss the opportunity to profit from chemotherapy. Therefore, it is essential to look for additional treatment strategies to improve the clinical efficacy and alleviate toxicity during platinum-based chemotherapy.

In China, traditional Chinese medicines (TCMs), as complementary and alternative medicines in patients with advanced NSCLC, play vital and positive roles in controlling tumor metastasis and decreasing toxicity as an adjunctive therapy, improving the cancer patients' immunity, the quality of life, and progression-free survival as a maintenance therapy, and providing a compelling therapeutic option as monotherapy [8–11]. Shenfu injection (SFI), which is extracted from two different Chinese herbs, Radix Ginseng and Radix Aconiti Lateralis Preparata, has been commonly used as an adjutant chemotherapy drug in China. A number of trials revealed that SFI had attenuation and synergistic efficacy to platinum-based chemotherapy [12, 13]. Our previous meta-analysis demonstrated that SFI combined with platinum-based chemotherapy might improve the quality of life, enhance the immune functions, and reduce adverse events compared with chemotherapy alone [14]. Nevertheless, the meta-analysis failed to manifest the benefit of SFI on the tumor response because of inconsistent or conflicting outcomes of the included studies, possibly caused by the limited quantity and quality of enrolled trials. Recently, there have emerged some new studies evaluating the efficacy of SFI combined with platinum-based chemotherapy for NSCLC. To shed light on these contradictory results and more precisely reveal its real synergistic efficacy and toxicity attenuation to platinum-based chemotherapy, we conducted this updated systematic review and meta-analysis to evaluate all related studies.

## 2. Methods

### 2.1. Literature Search Strategy

The following major Chinese or English language electronic databases including the PubMed, EMBASE, China National Knowledge Infrastructure Database (CNKI), the Cochrane Library, WanFang Database, and China Biological Medicine Database (CBM) were searched up to February 2017. Two reviewers (Ailing Cao and Hailang He) independently searched articles in electronic databases using the search strategy (Neoplasm [Mesh] OR Lung Neoplasm [Mesh] OR Pulmonary Neoplasms OR Pulmonary Neoplasm OR Lung Cancer OR Thoracic Neoplasm OR Pulmonary Cancer OR Lung Carcinoma OR Pulmonary Carcinoma OR NSCLC OR Non-small Cell Lung Cancer) AND (Shenfu OR Shenfu injection). All retrievals were implemented by the Mesh and free word.

### 2.2. Inclusion Criteria

Included studies must meet the following criteria: the disease was diagnosed and confirmed with NSCLC by histopathological or cytological diagnostic criteria. The stage of NSCLC TNM was advanced stage (III-IV). Type of study was randomized controlled trial (RCT). The patients of each study were divided into two arms. The intervention of one arm was platinum-based chemotherapy alone, whereas the intervention in the other arm was platinum-based chemotherapy plus SFI. Moreover, the reported data must have at least one of following outcomes: (1) objective tumor response (ORR); (2) disease control rate (DCR); (3) Karnofsky performance score (KPS); (4) grade 3 or 4 white blood cell, platelet, hemoglobin, and vomiting toxicity; (5) relevant indicators of cellular immune function: percentages of total T lymphocytes (CD3^+^), helper T lymphocytes (CD4^+^), and helper T lymphocytes (CD4^+^)/cytotoxic T lymphocytes (CD8^+^). The reported data also needed to have sufficient details to permit the calculation of the risk ratios and its 95% CIs for each outcome.

### 2.3. Exclusion Criteria

Relevant clinical trials were manually removed if any of the following factors was identified: (1) the studies that included patients with other malignancy; (2) the interventions that were combined with other Chinese herbs or other TCM therapies; (3) duplicated articles; (4) the design scheme of the research was not clear, or the data was not complete.

### 2.4. Outcome Measures

Outcome measures included primary and secondary indices. ORR and DCR were primary outcomes. KPS, adverse effects of white blood cell, platelet, hemoglobin, and vomiting toxicity and the percentages of CD3^+^, CD4^+^, and CD4^+^/CD8^+^ were regarded as the secondary indices of evaluation. ORR, formulated by the WHO scale [15], equals complete response (CR) + partial response (PR) and DCR equals complete response (CR) + partial response (PR) + no change (NC). The KPS [16] was employed to investigate the performance status of patients in many of the included studies which applied a 10-point change as the cutoff for improved or worse performance status. Therefore, we calculated the improved performance status as the number of patients with improved performance status (>10-point increase) divided by the total. The 5-point WHO scale [15] was used to evaluate chemotherapy toxicity and the rate of severe chemotherapy toxicity was defined as the number of patients with any severe toxicity (WHO grade 3 or 4) divided by the total number of patients in each treatment group (WHO grades 0, 1, 2, 3, and 4). Relevant indicators of cellular immune function including CD3, CD4^+^, and CD4^+^/CD8^+^ were also used to assess the efficacy of SFI.

### 2.5. Data Extraction and Quality Assessment

Two investigators (Ailing Cao and Hailang He) reviewed the eligible studies and extracted the data independently. This course had to be cross-checked in order to ensure accuracy and reliability. Any discrepancy was resolved by consultation of the 3rd reviewer (Xianmei Zhou). The required information was collected from each article: (1) basic information such as language, year of publication, and name of the first author; (2) number of participants, sex, age, physical status, and TNM stage information in each group; (3) details of interventions and outcomes from each studies. The methodological quality of the included RCTs was assessed independently by two reviewers (Ailing Cao and Hailang He) based on the criteria in the Cochrane evaluation handbook of RCTs 5.1.0 [17]. Briefly, the main questions about quality were (1) sequence generation; (2) allocation concealment; (3) blinding of participants and study personnel, blinding of outcome assessments; (4) incomplete outcome data: including baseline measurements before the intervention and effect parameters after intervention, dropout/exit (whether the dropout rate is less than 10%); (5) selective outcome reporting. Each term was identified as having low, unclear, or high risk of bias according to information provided by the protocol.

### 2.6. Statistical Analysis

The RevMan 5.3 software (Cochrane Collaboration) was used to perform the meta-analysis. The weighted mean differences (WMD) and relative risk (RR) with 95% confidence were calculated to compare continuous and dichotomous variables, respectively. If the heterogeneity existed in pooled studies (*I*^2^ > 50%), the random model was applied. Otherwise, the fixed model was used. Statistical significant difference was considered as *P* < 0.05. Funnel plots were employed to evaluate the potential publication bias for primary outcomes if more than 10 studies were included for a meta-analysis [17]. Publication bias was further evaluated by Egger's test with Stata 12.1 software.

### 2.7. Sensitivity Analysis

In this study, sensitivity analysis was employed to verify the robust and reliable results from our study. We conducted the analysis by deleting the poor studies with relatively high overall risk of bias.

## 3. Results

### 3.1. Retrieval Result

**Figure 1:**
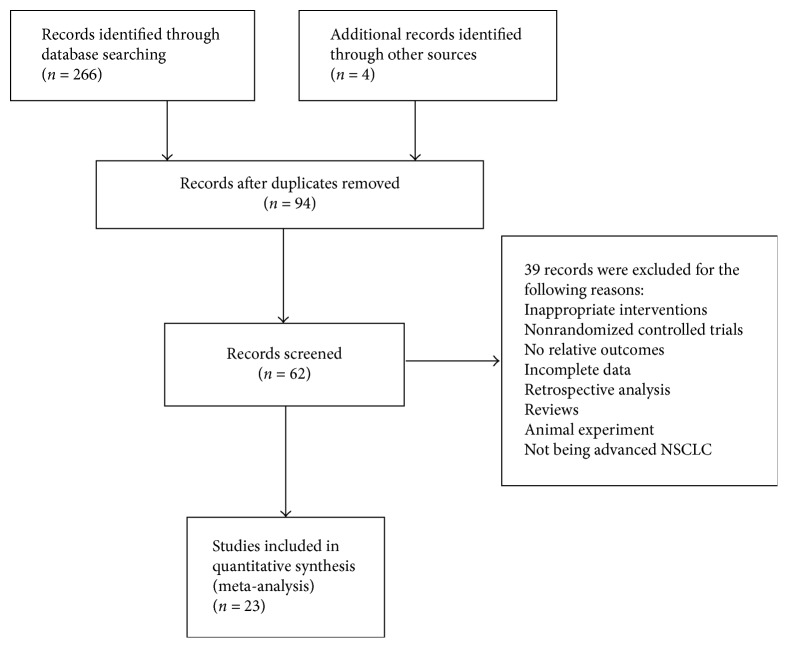
Flow diagram of the literature search process.

The initial database search identified 270 potentially relevant possible studies by using our search strategies from electronic database searching. A total of 62 records were identified after removing duplicates and screening the titles and abstracts. 39 trials were excluded with the following reasons: animal experiment (*n* = 3), reviews (*n* = 9), inappropriate interventions (*n* = 10), retrospective study (*n* = 1), non-RCTs (*n* = 8), no relative outcomes (*n* = 3), incomplete data (*n* = 2), and not being advanced NSCLC (*n* = 3). 23 clinical trials were finally involved in this meta-analysis. A flow diagram describing literature search and study selection was shown in Figure 1.

### 3.2. Characteristics of Included Trials

Table 1 presents a summary of the baseline characteristics of the enrolled studies, including authors, published years, number of cases, the performance status, TNM stage information, details of interventions, and outcomes. As shown, all of the studies were conducted in China and published in Chinese journals. The dosage of SFI was 30–100 ml per day and the duration of therapy was 1–3 weeks and 2–4 cycles by intravenous injection. NP regimen was the most common chemotherapy regimen [23, 27–29, 31, 32, 36, 38, 40], and the remainder included GP, TP, DP, GC, and TC regimens that were applied in 6 [19, 20, 24–26, 34], 4 [21, 30, 37, 39], 2 [18, 22], 1 [33], and 1 [35] studies, respectively. The stages of NSCLC TNM of the patients enrolled in the present studies were all advanced stage.

### 3.3. Methodological Bias of the Included Studies

**Figure 2:**
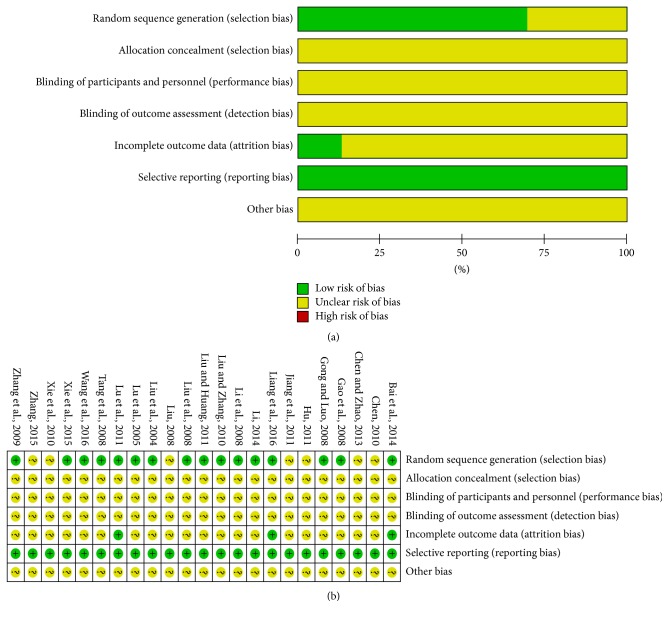
*Risk of methodological bias of the included studies*. (a) Risk of bias graph: review authors' judgments about each risk of bias item presented as percentages across all included trials. (b) Risk of bias summary: review authors' judgments about each risk of bias item presented as percentages across all included trials.

All of the included trials mentioned randomization, but only 16 [18, 19, 21–23, 25, 28–30, 33, 35–40] described the specific method of randomization. After attempts verification by contacting the authors of the original papers through phone or e-mail, 16 [18, 19, 21–23, 25, 28–30, 33, 35–40] trials were randomized by using random number tables to generate a sequence. Two [20, 24] of the remaining trials also were randomized by using the same methods. Although much effort had been made to contact the original authors, we still failed to get in touch with 5 [26, 27, 31, 32, 34] of them. None of the trials mentioned allocation concealment methods. The blinding procedure was not mentioned in all studies. These indicated that there were the selective bias and high implementation bias. Three trials [18, 22, 29] showed the results of data integrity. Selective reporting did not appear in all of the studies. Other bias was not clear. The detailed information of methodological quality of the included studies is listed in Figure 2.

### 3.4. Meta-Analysis for Objective Tumor Response

**Figure 3:**
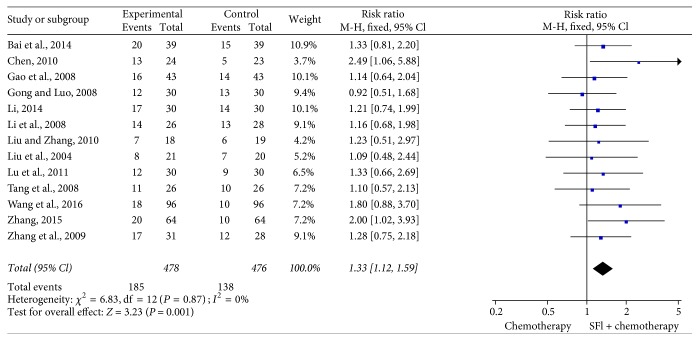
*Forest plot of improved objective tumor response*. Objective tumor response evaluated from meta-analysis of pairwise comparisons in patients with chemotherapy combined SFI versus chemotherapy alone.

13 trials [19, 20, 22, 23, 25, 29–31, 33, 35, 36, 38, 40] including 954 cases reported ORR (Figure 3). There was no significant heterogeneity among the trials (*I*^2^ = 0%, *P* = 0.87). Therefore, the fixed-effects model was applied for the analysis. The results of meta-analysis showed that the combination treatment of SFI and platinum-based chemotherapy significantly improved the objective tumor response of patients with NSCLC when compared with the chemotherapy alone [RR = 1.33, 95% CI (1.12, 1.59), *P* = 0.001].

### 3.5. Meta-Analysis for Disease Control Rate

**Figure 4:**
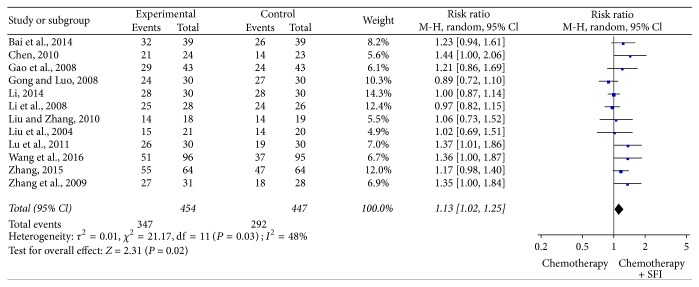
*Forest plot of improved disease control rate*. Disease control rate evaluated from meta-analysis of pairwise comparisons in patients with chemotherapy combined SFI versus chemotherapy alone.

DCR could be definitively extracted from twelve reports [19, 20, 22, 23, 25, 29–31, 33, 35, 38, 40]. There was heterogeneity between studies (*I*^2^ = 48%, *P* = 0.03). The pooled RR for DCR revealed that there was a remarkable improvement for the combination treatment of SFI and chemotherapy treatment yielding a RR of 1.13 [95% CI (1.02, 1.25), *P* = 0.02] by random-effects model (Figure 4).

### 3.6. Meta-Analysis for Improved KPS

**Figure 5:**
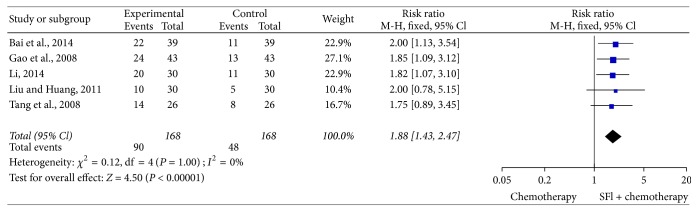
*Forest plot of improved KPS*. KPS evaluated from meta-analysis of pairwise comparisons in patients with chemotherapy combined SFI versus chemotherapy alone.

In 23 trials, 5 trials [22, 23, 25, 29, 35], including 336 cases, reported improvement rates of KPS (Figure 5). The heterogeneity test found that the data was homogeneous (*I*^2^ = 0%), employing the fixed-effects model in this meta-analysis. In meta-analysis, a statistically significant difference [RR = 1.88, 95% CI (1.43, 2.47), *P* < 0.00001] existed between SFI combination group and control group, which meant that a combination of SFI and chemotherapy could result in better quality of life.

### 3.7. Meta-Analysis for Chemotherapy Toxicity

#### 3.7.1. White Blood Cell

**Figure 6:**
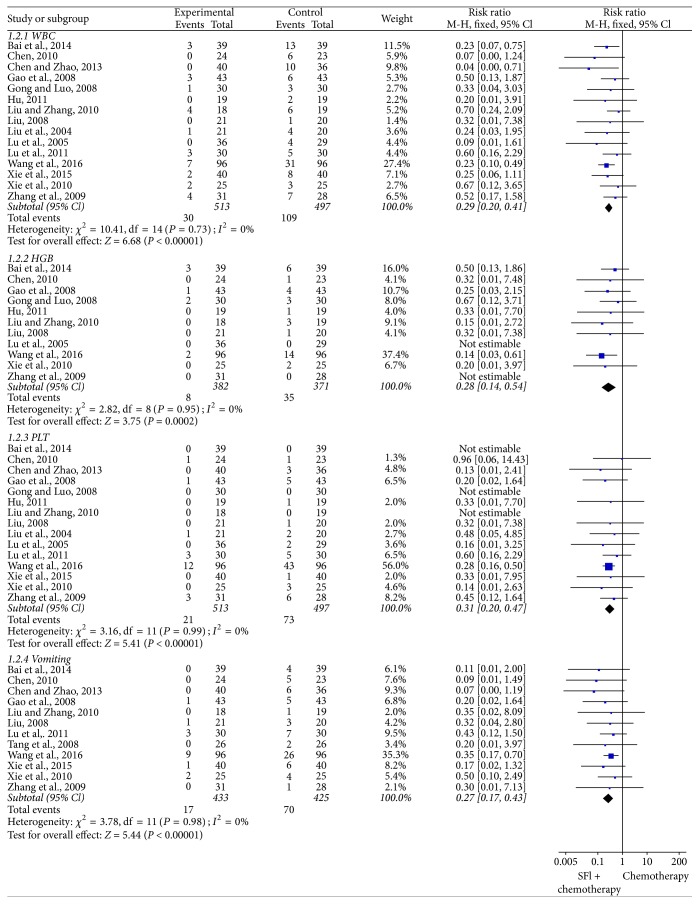
*Forest plot of chemotherapy toxicity*. White blood cell, hemoglobin, platelet, and vomiting toxicity evaluated from meta-analysis of pairwise comparisons in patients with chemotherapy combined SFI versus chemotherapy alone.

The incidence of white blood cell toxicity was reported in 15 trials [19, 21, 22, 24–26, 29–34, 38–40], which included 1010 patients (Figure 6). As the heterogeneity test showed *I*^2^ = 0%, a fixed-effects model was applied to calculate the combined RR and 95% CI. The results indicated that SFI group exhibited significant reduction in white blood cell toxicity compared with chemotherapy group alone [RR = 0.29, 95% CI (0.20, 0.41), *P* < 0.00001].

#### 3.7.2. Hemoglobin

**Figure 7:**
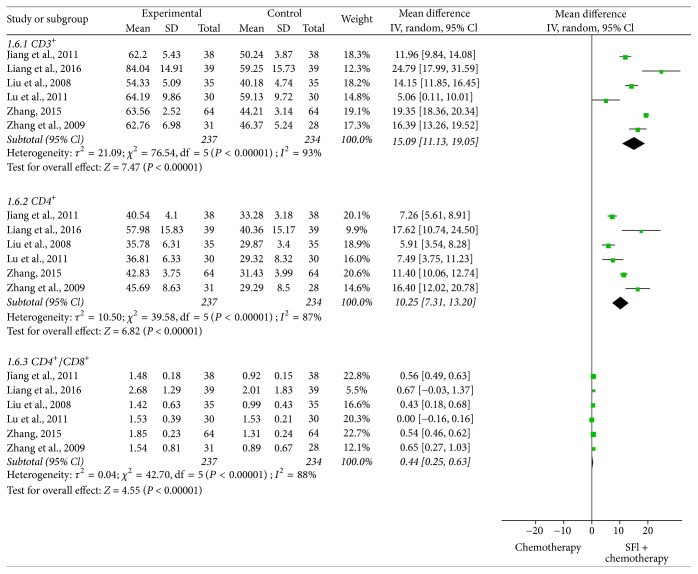
*Forest plot of immune function*. CD3^+^, CD4^+^, and CD4^+^/CD8^+^ evaluated from meta-analysis of pairwise comparisons in patients with chemotherapy combined SFI versus chemotherapy alone.

11 studies [19, 22, 24, 26, 30–34, 38, 39] provided data on the hemoglobin toxicity with 753 cases after treatment (Figure 6). There was no significant heterogeneity in the included trials (*I*^2^ = 0%). As shown in Figure 7, there was statistically significant lower severe toxicity for hemoglobin [RR = 0.28, 95% CI (0.14, 0.54), *P* = 0.0002] between the 2 groups by fixed-effects model, which suggested that SFI combined with chemotherapy could greatly decrease the rate of hemoglobin toxicity in the treatment of NSCLC when compared with chemotherapy alone.

#### 3.7.3. Platelet

Trials [19, 21, 22, 24–26, 29–34, 38–40] containing 1010 patients evaluated the platelet toxicity (Figure 6). It proved to be homogeneous according to the heterogeneity test (*I*^2^ = 0%), so the fixed-effects model effect model was used in this meta-analysis. As illustrated in Figure 6, combination treatment with chemotherapy plus SFI had an advantage in mitigating the toxicity of platelet compared to chemotherapy alone [RR = 0.31, 95% CI (0.20, 0.47), *P* < 0.00001].

#### 3.7.4. Vomiting

There were 12 trials [19, 21, 22, 24, 25, 29–34, 36] included in the meta-analysis with 858 patients of vomiting toxicity (Figure 6). The heterogeneity test was homogeneous (*I*^2^ = 0%), and fixed-effects model was applied in this pooled analysis. Meta-analysis revealed that SFI treatment could significantly reduce the incidence of vomiting toxicity than control group [RR = 0.27, 95% CI (0.17, 0.43), *P* < 0.00001].

### 3.8. Meta-Analysis for Immune Function

In all included studies, 6 trials [18, 20, 27, 29, 30, 37] containing 471 patients reported the percentages of CD3^+^, CD4^+^, and CD4^+^/CD8^+^ (Figure 7). According to the heterogeneity test, the random-effects model was used to calculate the combined WMD and 95% CI. Meta-analysis indicated that there was a statistically significant difference between two groups for CD3^+^ [WMD = 15.09, 95% CI (11.13, 19.05), *P* < 0.00001], CD4^+^ [WMD = 10.25, 95% CI (7.31, 13.20), *P* < 0.00001], and CD4^+^/CD8^+^ [WMD = 0.44, 95% CI (0.25, 0.63), *P* < 0.00001], which explained that SFI combined with chemotherapy could obviously enhance the immune function of NSCLC patients.

### 3.9. Analysis of Publication Bias

**Figure 8:**
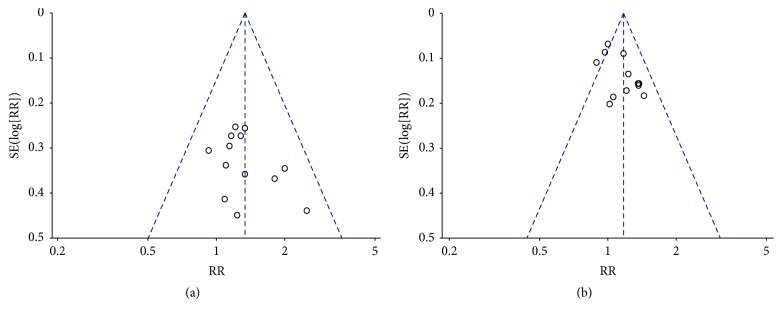
*The funnel plots for assessing publication bias*. (a) Objective tumor response. (b) Disease control rate.

Funnel plots and Egger's test were performed to identify potential publication bias among the included studies. The funnel plots were asymmetric in the studies about objective tumor response and disease control rate (Figures 8(a) and 8(b)), which showed that there was potential risk of publication bias. Not only would publication bias cause asymmetry in funnel plots, but clinical heterogeneity or methodology heterogeneity between studies might affect the shape of funnel plots. So Egger's test was further applied to evaluate publication bias. Egger's test for objective tumor response (*P* = 0.386) and disease control rate (*P* = 0.697) revealed that no proof of publication bias was obtained.

## 4. Sensitivity Analysis

The results of the fixed-effects and random-effects model had good consistency. After deleting the low quality studies with relatively high overall risk of bias, the results were still similar to the results before they were excluded (Table 2), which revealed that the results of our meta-analysis were reliable and verifiable.

## 5. Discussion

TCMs, used in Asia for centuries, are beginning to play a role in western health care as complementary and alternative medicines. For cancer patients, they can enhance efficacy and decrease toxicity when combined with radiotherapy and chemotherapy. As a traditional Chinese medicine injection, SFI, which consists of extract of Radix Ginseng and Radix Aconiti Lateralis Preparata (prepared aconite root), has been widely used in the treatment of NSCLC. The active ingredients of SFI are mainly ginsenoside, aconitine, and ginseng polysaccharide. Modern pharmacological researches have proved that the ginsenoside Rg3 plays an important role in suppressing tumor cell proliferation, inhibiting tumor cell invasion and metastasis, and promoting the apoptosis of tumor cells [41]. Aconitine has effects on inhibiting cell proliferation invasion and metastasis of human adenocarcinoma A549 cell lines through decreasing the expression of MMP-2 and MMP-9 activity [42]. Furthermore, it can reduce high KB_V200_ cell protein expression, partially reverse the cell resistance, and increase the sensitivity of chemotherapy drugs [43]. Ginseng polysaccharide has multiple immunomodulatory effects and inhibitive action on A549 cell growth and apoptosis promoting effect as well [44]. Consequently, SFI may significantly increase the clinical efficacy through inducing the cancer cell apoptosis, inhibiting cell proliferation metastasis, and upregulating tumor immunity.

In this study, 23 RCTs containing 1574 NSCLC patients were included, which ensured adequacy specimen for meta-analysis. Most of outcomes have good objectivity and stability. Prior to our meta-analysis [14], a meta-analysis included 19 studies was published in Chinese, but it did not reveal significant results of SFI on the ORR and there was no evaluation associated with DCR. Different inclusion criteria and the limited quantity may be responsible for the inconsistent findings between these two meta-analyses. Notably, as an updated systematic review with more data, the present results for ORR and DCR exhibited the same consistently superior effect of SFI combined with platinum-based chemotherapy in terms of efficacy enhancing for advanced NSCLC. Egger's test indicated that there was lower publication bias about ORR and DCR. After excluding the poor studies with relatively high overall risk of bias, meta-analysis showed that the results before and after exclusion had a good consistency. The above data suggested that SFI likely potentiated antitumor action of chemotherapy.

Adverse effects often occur in patients with advanced NSCLC when chemotherapy is used. The leukopenia, anemia, thrombocytopenia, and vomiting at toxicity grade of III-IV were clearly decreased in patients with SFI-containing treatments. The results were also in accordance with previous findings [14]. After deleting the low quality studies, the results were still similar to the results before they were excluded, which showed that the results had a good consistency and stability. Furthermore, the animal experimental study indicated that SFI could markedly enhance the level of WBCs, platelets, RBCs, and hemoglobin of myelosuppressed rats through promoting hematopoiesis [45]. So we believe that SFI plus chemotherapy can play a constructive role in reducing chemotherapy toxicity.

The toxicity of chemotherapy can impair immune function and lower the quality of life for NSCLC patients. The meta-analysis suggested that SFI could improve the quality of life. However, the results were limited by smaller samples in the meta-analysis of KPS. This might lead to insufficient assessment to them. Many experiments had reported that SFI could stimulate immune cells to enhance their anticancer activity [46, 47]. In this meta-analysis, we collected data relevant to immune function and pooled them together to find out whether SFI did have an effect on the cellular immunity of advanced NSCLC patients. The meta-analysis showed the percentages of CD3^+^, CD4^+^, and CD4^+^/CD8^+^ were significantly improved with a good consistency. The results seemed to indicate that SFI may exert its antitumor effect via enhancing the immune function of cancer patients. This conclusion needs to be confirmed by further large sample, high-quality RCTs and further investigation about underlying mechanisms of antitumor effect.

Although our meta-analysis demonstrates favorable outcomes in a combination of SFI and chemotherapy, several limitations in the present meta-analysis must be taken into account. Firstly, all the included trials manifested at least some methodological deficiencies leading to potential high risks of bias. The reporting of trial methods, procedures, and execution was frequently unclear, vague, and insufficient. After attempts verification by contacting the authors of the original papers through phone or e-mail, 18 trials were randomized by using random number tables to generate a sequence. We failed to get in touch with the remaining authors. None of included trials provided the detailed information on the concealment of treatment allocation and blinding. Therefore, there were potential risk of selection bias, performance bias, and detection bias in the present systematic review, which would lead to the overestimation of the clinical efficacy and attenuation of the treatment group. Secondly, all of the included studies were published in Chinese which might lead to ethical bias. Thirdly, the diversity of therapeutic dose, the small samples, and the lack of long-term follow-ups degraded the validity of the evidence of the clinical trials. Fourthly, all of the included studies applied an “A + B versus B” design where patients were randomized to accept SFI plus chemotherapy versus chemotherapy alone, without a rigorous placebo control. Such a design probably resulted in false positive results [48]. Altogether, the methodological quality of the included trials is insufficient and the potential benefits of SFI for NSCLC patients need to be further appraised through RCTs that employ rigorous methodology and include adequate assessment of the safety profiles of the interventions.

## 6. Conclusion

In summary, we find evidence that SFI combined with chemotherapy may amend tumor response, improve KPS, reduce chemotherapy toxicity, and enhance immune function when combined with chemotherapy alone in NSCLC patients. However, due to the poor quality of trials, the conclusion should be interpreted with caution. High-quality, precisely evaluated, and large sample size researches, particularly in the descriptions of methodology and study processes, are urgently needed to support the conclusion.

## Figures and Tables

**Table 1:** Baseline characteristics of included studies.

Study	*N* (T/C)	Physical	Stage	Interventions		Outcomes
				*T*	*C*	
Liang et al., 2016 [18]	39/39	KPS ≥ 60	III-IV	DP + SFI (80 ml/d, d1–d10)	DP	⑦
Wang et al., 2016 [19]	96/96	PS ≤ 2	IIIb-IV	GP + SFI (100 ml/d, d1–d15)	GP	①②③④⑤
Zhang, 2015 [20]	64/64	NR	III-IV	GP + SFI (60 ml/d, d1–d21)	GP	①⑦
Xie et al., 2015 [21]	40/40	PS ≤ 2	III-IV	TP + SFI (80 ml/d, d1–d7)	TP	②④⑤
Bai et al., 2014 [22]	39/39	KPS ≥ 60	III-IV	DP + SFI (80 ml/d, d1–d10)	DP	①②③④⑤⑥
Li, 2014 [23]	30/30	PS ≤ 2	III-IV	NP + SFI (50 ml/d, d1–d10)	NP	①⑥
Chen and Zhao, 2013 [24]	40/36	KPS ≥ 60	IIIb-IV	GP + SFI (30 ml/d, d1–d14)	GP	②④⑤
Gao et al., 2008 [25]	43/43	KPS ≥ 60	III-IV	GP + SFI (60 ml/d, d1–d10)	GP	①②③④⑤⑥
Hu, 2011 [26]	19/19	PS ≤ 2	III-IV	GP + SFI (60 ml/d, d1–d9)	GP	②③④
Jiang et al., 2011 [27]	38/38	KPS ≥ 60	III-IV	NP + SFI (60 ml/d, d1–d10)	NP	⑦
Liu and Huang, 2011 [28]	30/30	KPS ≥ 70	III-IV	NP + SFI (60 ml/d, d1–d14)	NP	⑥
Lu et al., 2011 [29]	30/30	KPS ≥ 60	III-IV	NP + SFI (60 ml/d, d1–d10)	NP	①②④⑤⑦
Liu and Zhang, 2010 [30]	18/19	PS ≤ 2	IIIb-IV	TP + SFI (60 ml/d, d1–d14)	TP	①②③④⑤
Chen, 2010 [31]	24/23	NR	III-IV	NP + SFI (50 ml/d, d1–d10)	NP	①②③④⑤
Xie et al., 2010 [32]	25/25	KPS ≥ 60	III-IV	NP + SFI (50 ml/d, 1–d10)	NP	②③④⑤
Zhang et al., 2009 [33]	31/28	KPS ≥ 60	IIIb-IV	GC + SFI (60 ml/d, d1–d10)	GC	①②③④⑤⑦
Liu, 2008 [34]	21/20	KPS ≥ 60	III-IV	GP + SFI (50 ml/d, d1–d10)	GP	②③④⑤
Li et al., 2008 [35]	26/28	KPS ≥ 70	IIIb-IV	TC + SFI (100 ml/d, d1–d7)	TC	①
Tang et al., 2008 [36]	18/19	KPS ≥ 60	IIIb-IV	NP + SFI (60 ml/d, d1–d7)	NP	①⑤⑥
Liu et al., 2008 [37]	35/35	PS ≤ 2	IIIb-IV	TP + SFI (40–60 ml/d, d1–d10)	TP	⑦
Gong and Luo, 2008 [38]	30/30	KPS ≥ 50	IIIb-IV	NP + SFI (50 ml/d, d1–d14)	NP	①②③④
Lu et al., 2005 [39]	36/29	KPS ≥ 60	IIIb-IV	TP + SFI (50 ml/d, d1–d14)	TP	②③④
Liu et al., 2004 [40]	21/21	KPS ≥ 60	IIIb-IV	NP + SFI (50 ml/d, d1–d14)	NP	①②④

*N*, number of participants; *T*, treatment; *C*, control; KPS, Karnofsky performance score; PS, performance status; SFI, Shenfu injection; NR, not reported; DP, docetaxel + platinum; GP, gemcitabine + platinum; TP, taxol + platinum; NP, navelbine + platinum; GC, gemcitabine + cisplatin; TC, taxol + cisplatin; ① objective tumor response; ② white blood cell toxicity; ③ hemoglobin toxicity; ④ platelet toxicity; ⑤ vomiting toxicity; ⑥ KPS; ⑦ immune function.

**Table 2:** Sensitivity analysis of this study.

Outcomes	*N*	RR or WMD (95% CI)	*I*^2^	Excluded the studies	*N*	RR or WMD (95% CI)	*I*^2^
ORR	13	1.33 (1.12, 1.59)	0%	[20, 31]	11	1.23 (1.02, 1.48)	0%
DCR	12	1.17 (1.08, 1.27)	48%	[20, 31]	10	1.15 (1.05, 1.26)	51%
WBC	15	0.29 (0.20, 0.41)	0%	[24, 26, 31, 32, 34]	10	0.32 (0.22, 0.48)	0%
HBG	11	0.28 (0.14, 0.54)	0%	[26, 31, 32, 34]	7	0.28 (0.13, 0.59)	0%
PLT	15	0.31 (0.20, 0.47)	0%	[24, 26, 31, 32, 34]	10	0.32 (0.20, 0.50)	0%
Vomiting	12	0.27 (0.17, 0.43)	0%	[24, 31, 32, 34]	8	0.30 (0.17, 0.50)	0%
CD3^+^	6	15.09 (11.13, 19.05)	93%	[20, 27]	4	14.75 (9.33, 20.17)	88%
CD4^+^	6	10.25 (7.31, 13.20)	87%	[20, 27]	4	11.36 (5.76, 16.97)	83%
CD4^+^/CD8^+^	6	0.44 (0.25, 0.63)	88%	[20, 27]	4	0.38 (0.22, 0.74)	82%

*Note*. WBC, white blood cell; HBG, hemoglobin; PLT, platelet; *N*, the number of trials.
